# Breakfast consumption habits of Australian men participating in the “Typical Aussie Bloke” study

**DOI:** 10.1186/s40795-019-0317-4

**Published:** 2020-01-07

**Authors:** Angelica Quatela, Amanda Patterson, Robin Callister, Lesley MacDonald-Wicks

**Affiliations:** 1 Discipline of Nutrition and Dietetics, School of Health Sciences, The University of Newcastle, Callaghan, NSW 2308 Australia; 2 Priority Research Centre for Physical Activity and Nutrition, The University of Newcastle, Callaghan, NSW 2308 Australia; 3 Hunter Medical Research Institute, New Lambton, NSW 2305 Australia; 4 School of Biomedical Sciences and Pharmacy, The University of Newcastle, Callaghan, NSW 2308 Australia

**Keywords:** Breakfast, Morning meal, Australia, Males, Young adults

## Abstract

**Background:**

Breakfast is often regarded as “the most important meal of the day” but there is limited reporting of the foods/beverages currently constituting a typical breakfast. This study investigated current breakfast habits of Australian men.

**Methods:**

Men aged 18-44y were recruited from metropolitan and regional NSW Australia and completed an online survey investigating breakfast consumption habits and other lifestyle parameters including demographic characteristics and waking habits.

**Results:**

112 men participated. Most (83.5%) ate breakfast ≥5 times/week and consumed this meal before 8 am (84.0%). Breakfast for habitual breakfast eaters consisted of one or more of the following foods or beverages eaten ≥5 times/week: breakfast cereal (50.0%), milk for cereal (51.1%), fruit (28.7%), toast (13.8%), spreads (11.7%), yogurt (12.8%), and/or coffee (40.4%). Breakfast may also include the following foods 1–4 times/week: eggs (58.5%), bacon (30.9%), juice (19.1%), and/or tea (17.0%).

**Conclusion:**

A majority of Australian men younger than 45 years old were found to eat breakfast most days of the week. Cereal, milk and fruit were the most common foods consumed for breakfast. Breakfast is considered to be an important meal among health professionals and we found a majority of Australian men do eat breakfast regularly. Approximately half of the young men in the study reported consuming cereal and milk for breakfast most of the time, a breakfast option that is linked to higher daily wholegrain, fibre and micro-nutrient intakes.

## Background

Breakfast is often referred to as the most important meal of the day [1] because it may contribute to better nutrient adequacy [2], which is believed to play a significant role in the prevention of chronic disease [3]. Also, breakfast is possibly protective against weight gain [1, 4]. The evidence available to support these beliefs, however, is limited and/or contradictory [1, 4–11].

There is a lack of current data regarding the consumption or skipping of breakfast, as well as the habitual foods or beverages that constitute a typical breakfast in a multicultural country like Australia. Prior to this study, the most recent data regarding Australian breakfast habits were reported by Williams [12] in a secondary analysis of 24-h recall data from the Australian National Nutrition Survey (NNS) in 1995 [12, 13]. Williams reported that cereal products, milk and fruit were the main foods consumed for breakfast by the majority of Australian adults but noted that there were differences between males and females in the foods and beverages consumed at breakfast, with a significantly lower percentage of men than women consuming fruit. As the 1995 NNS was conducted over 20 years ago, it is likely that the food habits of Australian men have changed. Therefore, the aim of the Typical Aussie Bloke study was to obtain current information regarding the breakfast habits and the food and beverage intakes that constitute a breakfast meal among a sample of < 45 y Australian men. Furthermore, the literature reported lack of evidence base regarding reasons for consuming breakfasts and timing of its consumptions. Therefore, his study adds to the current literature by examining the reasons for consuming or skipping breakfast and the timing of breakfast consumption.

## Methods

### Study design

Men aged 18-44y were recruited from metropolitan (Newcastle) and regional (Tamworth) areas in NSW Australia for a multicentre cross sectional study. The project was advertised using conventional (e.g., radio, television, magazines) and social (e.g.; university blogs, university Facebook) media, recruitment fliers and in person recruitment in key community locations (e.g., Callaghan campus, Tamworth Education centre, Hunter Medical Research Centre, gyms). Participants completed an online survey and underwent a measurement session in the laboratory at the University of Newcastle’s Callaghan Campus or Tamworth Education Centre. Participants provided implied consent for the online survey and written consent for the laboratory measurement session. The protocol was approved by the University of Newcastle’s Human Research Ethics Committee (H-2015-0199) and data were collected from September to December 2015.

### Inclusion and exclusion criteria

Participants were included if they were male and aged 18 to 44 years. The exclusion criteria were: being unable to attend a laboratory measurement session, having had cosmetic surgery that would change the shape of the body, having or being treated for a thyroid condition or insulin dependent diabetes, or claustrophobia. Participants were screened for eligibility by the first eight questions of the online survey.

### Survey

The online survey (Survey Monkey Inc., San Mateo, CA, USA) consisted of 56 questions. Following the screening questions, eligible participants were directed to the remaining 48 questions which investigated parameters such as demographic characteristics, breakfast consumption habits and waking habits.

Standard demographic and dietary questions were used where possible and most were consistent with questions from the Australian Longitudinal Study of Women’s Health (ALSWH) [14] or Australian Bureau of Statistics [15] surveys. The question assessing habitual breakfast consumption was from the 1995 NNS [13] and details of the question can be found in Additional file 1 [13]. Participants were defined as Habitual Breakfast Eaters (HBE) if they consumed breakfast 5 or more times during the week, Occasional Breakfast Eaters (OBE) if they consumed breakfast 3–4 times a week or Habitual Breakfast Skippers (HBS) if they consumed breakfast 1–2 times a week or rarely/never. Habitual food and beverage intake for breakfast was defined as an intake of these items ≥5 times per week. This definition was used to identify the foods/beverages that comprised a typical breakfast for this cohort. Questions regarding reasons for consuming or not consuming breakfast were taken from Reeves et al. [16]. The questions can be found in Additional file 1.

Questions investigating the timing of waking, the timing of consumption of the first meal of the day, and the types of food and beverages consumed for breakfast were specifically developed for this study and were pilot-tested among staff at the University of Newcastle. The questions are described in Additional file 1.

The questions investigating foods and beverages consumed at breakfast were provided in the form of a short food frequency questionnaire (FFQ) and the questions are described in Additional file 1.

Only participants who reported consuming breakfast at least 1–2 times per week were asked to complete the questions about the types of food and beverages consumed, and reasons for consuming breakfast.

### Statistics

Statistics were performed using Stata 13.1 (StataCorp LLC, College Station, Texas, USA). Descriptive statistics, such as means and distributions were calculated, and inferential statistics, including Chi Square or Fishers Exact tests, one way ANOVA or independent t test, were used to compare HBE versus OBE versus HBS, and for HBE consuming this meal early (EBE) versus late (LBE).

## Results

### Demographic characteristics

Data were obtained from 112 men, 87 subjects were from Newcastle and 25 from Tamworth. The majority of participants were born in Australia (87.5%) and only one (0.9%) was of Aboriginal or Torres Strait islander descent. A majority of participants were married or in a de facto relationship (51.7%) and had a university qualification (56.3%). Most (55.3%) were in full time employment, 25% were studying, and only a few men (2.7%) were on home duties or unemployed. The distribution of income was wide with slightly more (55.4%) in the lower income brackets (ranging from ≤AUS$25,000 to <AUS$99,000 per annum). Most lived with one or more other people (88.4%) and did not have any dependent children (70.5%). The majority of the participants were employed as professionals, paraprofessionals, managers or administrators, administrative assistants, sales or personal service workers (88.4%); only a minority worked in trades or as manual workers, machine operators or drivers (9.8%).

### Comparison of breakfast consumption patterns

The majority of men were HBE (83.9%, *n* = 94) consuming breakfast ≥5 times a week. Only a small proportion of participants were HBS (9.3%, *n* = 10) consuming breakfast either never, rarely or 1–2 times per week. The other participants were OBE (7.8%, *n* = 7) who consumed breakfast 3–4 times a week; one person did not report his frequency of breakfast consumption.

Table 1 describes the characteristics of HBE (*n* = 94), OBE (*n* = 7) and HBS (*n* = 10). These three groups were similar across all demographic characteristics except level of education. HBE were significantly more likely to have tertiary qualifications (62.8%), while a higher percentage of OBE (71.4%) and HBS (80.0%) had only secondary school qualifications (*p* = 0.010).

**Table 1:** Demographic characteristics of participants of the Typical Aussie Bloke study categorised by habitual breakfast eating patterns

	Habitual Breakfast Eaters (HBE) (*n* = 94)	Occasional Breakfast Eaters (OBE) (n = 7)	Habitual Breakfast Skippers (HBS) (*n* = 10)	*P* value
% (n)	83.9% (94)	6.3% [7]	8.9% [10]	
Age y (mean ± SD)	30 ± 7	25 ± 5	31 ± 6	0.19
^a^Height (mean ± SD)	179.8 ± 6.6	177.0 ± 5.0	180.5 ± 5.3	0.48
^a^Weight (mean ± SD)	82.5 ± 14.7	80 ± 14.4	84.2 ± 11.7	0.84
^a^BMI (mean ± SD)	25.5 ± 4.2	25.5 ± 3.8	26.0 ± 4.3	0.95
Country of birth				
Australia	86.2%	100.0%	100.0%	
^b^Other countries	13.8	0	0	0.47
ATSI descent				
Yes	0%^*^	0%	10.0%	
No	98.9^*^	100.0	90.0	0.16
Marital status				
^c^Single	44.7%^*^	42.9%	70.0%	
Married or de facto relationship	54.3^*^	57.1	30.0	0.35
Education				
^d^High school or trade	36.2%^*^	71.4%	80.0%	
^e^University or higher degree	62.8^*^	28.6	20.0	*0.010*
Employment status				
Full time paid work	53.2%	57.1%	80.0%	
Part time paid work or casual paid work	18.1	14.3	0	
Studying	25.5	28.6	20.0	
Home duties or Unemployed	3.2	0	0	0.77
Annual household gross income				
≤ AUS$25,000 to 49,999	31.9%^*^	14.3%	20.0%	
≥ AUS$50,000 to 99,999	21.3^*^	57.1	50.0	
≥ AUS$100,000 to 149,999	21.3^*^	14.3	20.0	
≥ AUS$150,000	20.2^*^	14.3	10.0	0.39
People living in the household				
Living alone	7.5%^*^	14.3%	20.0%	
^f^Living with other people	90.4^*^	85.7	80.0	0.23
Dependent children				
None	69.2	100.0	70.0	
One or more	30.9	0	30.0	0.26
Past/present or Future Occupation				
Trades, manual workers, machine operators or drivers	8.5%	0%	30.0%	
Professional, Paraprofessional, managers or administrator, administrative assistant, sales or personal service worker	90.4	100.0	70.0	
Never had a paid job	1.1	0	0	0.24

*The total number of subjects for this variable do not add up to 100% due to missing data reported (such as replied: ‘prefer not to answer’ or ‘don’t know’ Or don’t know/Varies’ or ‘don’t know or would rather not say’)

Please also note: one participant was excluded from the data displayed in this table because it was not possible to classify his breakfast consumption habits as he answered ‘do not know/varies’ to the Breakfast Consumption Habits question

*HBE* Habitual Breakfast Eaters who reported consuming breakfast 5 or more times per week

*OBE* Occasional Breakfast Eaters who reported consuming breakfast 3 to 4 times per week

*HBS* Habitual Breakfast Skippers who reported consuming breakfast ‘rarely or never’ or 1 to 2 times per week’

^a^Height, weight and BMI were measured in the lab session

^b^Others country: New Zealand, Italy, Germany, United Kingdom, Netherlands, America, Canada, Bangladesh or South Korea or unknown

^c^Single includes: separated, divorced, widowed or never married

^d^School includes: Intermediate Certificate (or equivalent) or Higher School or Leaving Certificate (or equivalent) or Trade/apprenticeship (eg. Hairdresser, Chef) or Certificate/diploma (eg. Child Care, Technician)

^e^University includes: University degree or University Higher degree (eg. Grad Dip, Masters, PhD)

^f^Living with other people includes living with one, two, three, four or five other people

ATSI – Aboriginal or Torres Strait Islander

^g^Statistics: One Way anova was used to compare age, height, weight and BMI. Chi Square tests and/or Fishers exact tests were used for the following comparison: Country of birth, ATSI descent, Marital status, Education, Employment status, people living in the household, Dependent children, Past,present and future occupation

### Breakfast patterns among habitual breakfast eaters

Table 2 shows the habitual and less frequent breakfast eating patterns amongst HBE. Coffee was the beverage most habitually consumed by these men (40.4%). Breakfast cereal (50%) with milk was the food option consumed most regularly. The most popular types of breakfast cereals consumed were: muesli (16.8%); cereals like Weet Bix, Vita Brits, Weeties (15.9%); and porridge (12.1%). Fruits were consumed regularly by 28.6% of the study participants. Toast (13.8%), spreads (11.7%), yoghurt (12.8%) and eggs (11.7%) were consumed regularly by smaller proportions of the study sample. With regards to the combination of foods, the highest frequency of consumption was breakfast cereal with milk and fruit (11.7%).

**Table 2:** Habitual and less frequent food and beverage consumption for breakfast among Habitual Breakfast Eaters (HBE)

	HBE consuming these foods/ beverages ≥ 5 times a week (*n* = 94)	HBE consuming these foods/beverages 1–4 times a week (*n* = 94)
Foods		
Any breakfast cereal	50.0%^a^	34.4%^b^
• Cereals like muesli	19.2%	22.3%
• Cereals like Weet Bix,Vita Brits, Weeties	17.0%	25.5%
• Cereals like All Bran	6.4%	5.3%
• Cereals like Cornflakes, Nutrigrain, Special K	4.3%	14.9%
• Cereals like Sultana bran, Fiber Plus and Branflakes	2.1%	4.3%
• Cereals like porridge	12.8%	24.5%
Milk for cereal	51.1%	21.3%
Fruit	28.7%	30.9%
Yogurt	12.8%	24.5%
Toast	13.8%	47.9%
Spread (e.g. jam, honey, peanut butter, Nutella, vegemite, etc.)	11.7%	36.2%
Butter and/or margarine	8.5%	41.5%
Eggs	11.7%	58.5%
Bacon	0	30.9%
Beans	0	13.1%
Pancakes	0	8.5%
Beverages		
Coffee	40.4%	18.1%
Tea	6.4%	17.0%
Juice	5.3%	19.1%
Smoothies	4.3%	11.7%
Milk on its own	1.1%	6.4%
Combinations of foods and/or beverages		
Cold breakfasts		
Any cereal & yogurt	10.6%	14.9%
Fruit & yogurt	7.4%	12.8%
Cereal with milk & fruit	11.7%	10.6%
Cereal, fruit & yogurt	6.4%	8.5%
Toast & butter or margarine & spread	5.3%	29.8%
Cooked breakfasts		
Toast & eggs	3.2%	36.2%
Bacon & eggs	0	26.6%
Toast, eggs, butter or margarine	0	29.4%
Toast, bacon & eggs	0	17.0%
Toast (and butter/margarine) with bacon and eggs.	0	13.8%

*HBE* Habitual Breakfast Eaters who reported consuming breakfast 5 or more times per week

^a^Represents participants who reported consuming one or more breakfast cereal types habitually; these participants could also have consumed one or more breakfast cereal types 1–4 times a week

^b^ Represents participants who consumed one or more breakfast cereals 1–4 times a week but did not consume one or more of the breakfast cereal types ≥5 times a week

### Less frequent breakfast patterns among habitual breakfast eaters

The most frequent foods and/or beverages consumed 1 to 4 times a week by HBE were eggs (58.5%), breakfast cereal (34.4%) and toast (47.9%). Butter or margarine was consumed by 41.5% and spreads by 36.2%, and bacon and fruits were each consumed by 30.9% of HBE. With regards to beverages, juice (19.1%) was the most popular drink, with coffee (18.1%) the second most popular beverage consumed 1 to 4 times a week. With regards to combinations of foods consumed < 5 times a week, popular combinations were: eggs and toast (36.2%) and bacon and eggs (26.6%). A less frequent combination was breakfast cereal with yoghurt (14.9%).

### Other foods and beverages consumed for breakfast by habitual breakfast eaters

Six men reported consuming shakes (e.g., protein shakes, green infusions, meal shakes or unspecified type). Four participants reported consuming servings of meat (ham, steak or chicken) and six men reported consuming vegetables (such as mushroom, zucchini, spinach, carrots, capsicum, celery or onion). Five participants reported consuming almond or coconut milk and six reported consuming either oats or nuts. Two men reported consuming cheese and two reported consuming English muffins. The number of times these foods/beverages were consumed in a usual week and whether this reached habitual consumption is unknown.

### Early vs late breakfast consumption and the relationship with waking habits

The majority of participants reported waking between 5.01 and 8.00 am during the week (all participants: 86.6%; HBE: 77.6%) and between 6.01 and 9.00 am during the weekend (all participants: 78.6%; HBE: 79.8%). Most HBE consumed the first meal of the day between 6.01 and 8.00 am (76.6%) during the week and 7.01 and 9.00 am on weekends (65.9%). Most HBE consumed the first meal of the day early during the week (84.0%) compared to the weekend (54.6%). Also, most HBE consumed their first meal of the day within two hours of waking during the week (86.2%) and the weekend (87.2%); only a small proportion consumed this meal between two and five hours after waking during the week (13.8%) or on the weekend (12.8%).

Table 3 compares the characteristics of HBE who consume the first meal of the day Early (*n* = 79) vs Late (*n* = 15) during the week. These two groups differed significantly with regards to age, marriage, income, and number of dependent children. EBE were significantly older (*p* = 0.0124) and more likely to be married (59.4%; *p* = 0.023) compared with LBE. A significantly higher percentage of EBE (69.8%) had a full-time job and earned ≥ AUD50K (69.7%) compared with LBE who were more likely to have a part time job (40.0%; p = 0.01) and earn less ≤ AUD49,999 (60.0%; *p* = 0.043). A higher percentage of EBE (35.6%) had one or more dependent children compared with LBE (6.7%, *p* = 0.032), however, in both groups the majority of men had no dependent children.

**Table 3:** Demographic characteristics of habitual breakfast eaters categorised by the timing of the first meal of the day

	Early Breakfast Eaters (EBE)^a^ (*n* = 79)	Late Breakfast Eaters (LBE)^b^ (*n* = 15)	P value
% (n)	84.0% (79)	16.0% [15]	
Age y (mean ± SD)	31 ± 7	26 ± 7	0.012
^c^Height (mean ± SD)	179.8 ± 6.3	179.8 ± 8.0	0.99
^d^Weight (mean ± SD)	81.9 ± 13.5	85.5 ± 20.4	0.38
^e^BMI (mean ± SD)	25.4 ± 4.1	26.5 ± 4.8	0.36
Country of birth			
Australia	86.1%	86.7%	
^f^Other countries	13.9%	13.3%	1.00
ATSI descent			
Yes	0^*^	0	
No	98.7%^*^	100.0%	NA
Marital status			
^g^Single	39.2%^*^	73.3%	
Married or in de facto relationship	59.4%^*^	26.7%	0.023
Education			
^h^School	31.6%^*^	60.0%	
^i^University	67.1%^*^	40.0%	0.076
Employment status			
Full time paid work	60.8%	13.3%	
Part time paid work or casual paid work	13.9%	40.0%	
Studying	24.1%	33.3%	
Home duties or Unemployed	1.3%	13.3%	0.001
Annual household gross income (AUS$)			
≤ $25,000 to 49,999	26.6%^*^	60.0%^*^	
≥ $50.000 to 99,999	24.1%^*^	6.7%^*^	
≥ 100.000 to 149,999	24.1%^*^	6.7%^*^	
$150,000 to 200,000 or more	21.5%^*^	13.3%^*^	0.043
People living in the household			
Living alone	6.3%^*^	13.3%	
^l^Living with other people	91.1%^*^	86.7%	0.32
Dependent children			
None	64.6%	93.3%	
One or more	35.4%	6.7%	0.032
Past/present or Future Occupation			
Trades, manual workers, machine operators or drivers	8.9%	6.7%	
Professional, paraprofessional, managers or administrator, administrative assistant, sales or personal service worker	89.9%	93.3%	
Never had a paid job	1.3%	0%	1.00

*The total number of participants for this variable does not add up to 100% due to responses such as ‘replied prefer not to answer’ or ‘don’t know’ Or don’t know/Varies’ or ‘don’t know or would rather not say’

*HBE* Habitual Breakfast Eaters who reported to consume breakfast 5 or more times per week

^a^*EBE* Early Breakfast Eaters includes Habitual Breakfast Eaters who consumed the first meal of the day early on weekdays (between 5.01 and 8.00 am)

^b^*LBE* Late Breakfast includes Habitual Breakfast Eaters who consumed the first meal of the day late on weekdays (between 8.01 am and noon)

*NA* Not Applicable

^c^Height, weight and BMI were measured in the lab session

^d^Others country: New Zealand, Italy, Germany, United Kingdom, Netherlands, America, Canada, Bangladesh or South Korea or unknown

Single includes: separated, divorced, widowed or never married.

^e^School includes: Intermediate Certificate (or equivalent) or Higher School or Leaving Certificate (or equivalent) or Trade/apprenticeship (eg. Hairdresser, Chef) or Certificate/diploma (eg. Child Care, Technician)

^f^University includes: University degree or University Higher degree (eg. Grad Dip, Masters, PhD)

^g^Living with other people includes living with one, two, three, four or five other people

*ATSI* Aboriginal or Torres Strait Islander

^h^Statistics: Indipendt t-test was used to compare age, height, weight and BMI. Chi Square tests and/or Fishers exact tests were used for the following comparison: Country of birth, ATSI descent, Marital status, Education, Employment status, people living in the household, Dependent children, Past,present and future occupation

### Reasons for consuming or not consuming breakfast

Reasons for consuming breakfast were provided by participants (*n* = 107) who reported consuming breakfast ≥1–2 times per week. The most common responses to the question investigating reasons for consuming breakfasts were: ‘I am hungry’ (30.8% (33)), ‘It is what I always do’ (24.3% (26)), ‘It gives me energy’ (19.7% [17]) or ‘It helps prevent me from getting hungry before lunch time’ (13.1% [14]). Fewer participants replied with the following: ‘I enjoy it’ (7.5% [8]), ‘Eating breakfast makes it easier to control my weight’ (1.9% [2]), ‘It helps me to wake up’ (0.9% [1]) or ‘Other reasons’ (2.8% [3]). None of the participants replied ‘I want to lose weight’ as a reason for consuming breakfast. Other reasons for consuming breakfast included ‘it is the most important meal of the day’, ‘to be social’ or ‘because the kids must eat breakfast’.

There were numerous reasons for not consuming breakfast. A majority of study participants reported that ‘I rarely/never don’t eat breakfast’ (52.7% (59)). Approximately 20% each of participants replied that: ‘I do not feel like eating first thing’ (18.0% [17]) or ‘Not enough time’ (17.0% [18]). A small number answered: ‘I do not have any food in the house’ (2.7% [3]), ‘I have a cigarette instead’ (1.8% [2]), ‘Hung over’ (0.9% [1]), ‘I want to lose weight’ (0.9% [1]) or other reasons (6.3% [7]). None of the participants replied ‘I do not have money for consuming breakfast’ as a reason for skipping breakfast. ‘Other reasons’ for not consuming breakfast included ‘having slept in’, ‘not wanting to eat before exercising’ or ‘skipping breakfast to do something different’.

## Discussion

This study investigated the habitual breakfast habits, reasons for consuming or skipping breakfast, and the timing of breakfast consumption for a sample of Australian men < 45 y. This study also collected current information regarding the habitual foods and beverages that constitute a breakfast meal among these men. In this study the majority of the participants were Habitual Breakfast Eaters and consumed this meal earlier than 8.00 am. Those who habitually ate breakfast early were more likely to have a university qualification, have higher incomes, dependent children, full-time jobs and be married/defacto than those who ate breakfast after 8 am. Breakfast cereal and milk formed the most frequent foods consumed habitually and coffee was the most common beverage consumed regularly. The main reasons for consuming breakfast were feeling hungry, needing energy, and it being a habit, whereas not having enough time or not feeling hungry were the main reasons for skipping breakfast.

In the Typical Aussie Bloke study there was a higher percentage of men (83.9%) who reported consuming breakfast habitually than in the 1995 NNS (73.5% of men aged ≥19 y; 57.5% of men aged 19–24 y; 66.5% of men aged 25–44 y [13]). The Typical Aussie Bloke study recruited a relatively small sample of men living in Newcastle and Tamworth whereas the NNS represented the breakfast habits of a much larger sample from across Australia (13,858 participants aged two or more years old from urban and rural areas in all States and Territories;). Thus, we would expect some differences based on sampling but there is also the possibility that breakfast habits have changed since 1995. This idea is supported by the findings of the OXFAM international survey in 2011 which reported that dietary changes occur over time. They found that in Australia, 62% of participants reported they had changed their eating habits in the previous two years [19].

There was a higher proportion of Australian men in the Typical Aussie Bloke study who reported being HBE compared with adults in other countries. In an American population of men aged 18 y and older (surveyed 1989–1991) 74.8% were breakfast consumers [20], and in an Italian population 73.6% of students consumed breakfast [18]. However, the proportion of HBE in this study was similar to the 89% of breakfast eaters in a Canadian population. The study of American men in 1989–1991 may not represent current eating habits [20]. Similarly, in the Italian study, although more recent, only undergraduate students aged 18–25 y were recruited and this may not be representative of the Italian population as a whole [18]. It is also important to note that these studies did not assess the regularity of breakfast consumption but only the percentage of people consuming breakfast on the day that the 24 h recalls were collected.

There are only a limited number of studies that have investigated the breakfast habits of people in Australia and other countries. Interestingly, the most common foods and beverages consumed by this sample in 2015 are similar to the most frequent foods and beverages consumed in the 1995 NNS. The most frequently consumed foods/beverages in 1995 by Australian men over 19 years old were: cereal products (76.2%) including cold (44.3%) and hot cereals (4.9%), breads (44.0%) and pastries/cakes/biscuits (44.0%), milk (65.6%) and tea or coffee (57.5%). Fruit was consumed by a smaller proportion of men (14.3%). Therefore, cereal, milk and fruit were the most common foods consumed in both studies, a breakfast option that is linked to higher daily wholegrain, fibre and micro-nutrient intakes. However, there appear to be some differences in the proportions of consumption of these common foods, for example, in fruit and cereal consumption. We must recognise the inherent differences in data collection and design between the studies and not draw any strong conclusions about changes in breakfast consumption over time, however, there is an indication that the proportion of men consuming fruit and breakfast cereal may have increased. The 1995 NNS assessed the foods/beverages consumed at breakfast on the day before data collection using a 24-h recall whereas our study assessed the number of times men consumed these foods/beverages at breakfast in a usual week using an FFQ, and was purposefully trying to ascertain routine breakfast food habits.

The breakfast foods consumed by Australian men were similar to the American men in the Continuing Survey of Food Intake, which included bread (19.5%), eggs (17.5%), ready to eat cereal (16.7%), fruits (4.8%) and cooked cereals (4.0%) [17]. However, Australian food choices for breakfast were considerably different to the Italian adults’ breakfast foods which included coffee (93.3%), sugar (81.6%), crispbread/rusk (87.9%), milk (67.7%), biscuits (42.9%), yogurt (42.8%), brioche (38%), jam (37.3%) and tea (33.4%) [21].

In this study, the HBE group differed in level of attained education, but no other factors were significantly different from OBE and HBS groups. This differs from the findings of the NNS 1995, where habitual breakfast consumption differed by income level (Breakfast eaters: Quintile 1 (lowest income) 83.9%, Quintile 5 (highest income) 79.7%; *p* < 0.005) [12]. The findings from the 1995 NNS found that people with the lowest incomes had a significantly higher proportion of HBE compared with people from all other income groups [12]. This difference could be due to the smaller sample size in the Typical Aussie Bloke study and the differences in sampling processes.

Interestingly, significant demographic differences were found between HBE who consumed the meal early (*n* = 79) and late (*n* = 15) during the week. The findings show that EBE could be considered to have higher levels of responsibilities, including being married, having a full-time job, earning a higher income and having dependent children, which most likely requires an organised consistent schedule early in the morning. LBE were more likely to be younger and single, have no dependent children and a lower income, which suggests that they had fewer responsibilities and therefore were less likely to follow a consistent schedule in the morning. Our study shows that among people who consumed breakfast habitually, breakfast was mostly consumed earlier than 8.00 am (84%) during the week, while a considerable proportion of HBE consumed this meal later than 8.00 am on the weekend (45.6%). This suggests that the timing of breakfast consumption is related to their commitments. Participants have breakfast earlier during the week because they may either have to go to work or to take their children to school or they have to study whereas during the weekend the majority of participants have breakfast later than 8.00 am as they are free from these commitments.

Habit, hunger and energy needs were the main drivers for the consumption of breakfast whereas lack of time and not feeling like eating first thing in the morning were the main reasons for not consuming breakfast. These findings are similar to those of Reeves et al. (2013) in the UK amongst 1068 subjects, as a large percentage of the participants replied ‘it gives me energy’ (70.2%), ‘I am hungry’ (66.1%) and/or ‘It is what I always do’ (57.4%) as the reasons for consuming breakfast [16]. Also, large proportions of the population replied ‘not enough time’ (40.2%) and ‘I do not feel the need to eat in the morning’ (49.5%) as the reasons for skipping breakfast [16].

This study has a number of strengths. This paper investigated timing of breakfast consumption and its relation to waking habits, which has not previously been reported in Australia. This is the first study to report data regarding the current habitual (2015) breakfast habits of men in Australia as there have been no other investigations of habitual breakfast consumption in Australian men reported since the 1995 NNS. There has been a more recent national nutrition survey (2011/12) in Australia but it did not investigate habitual breakfast patterns so was not used for comparison with this study [22]. For our study, we used a diverse range of recruitment strategies (fliers, conventional and social media) and multiple data collection sites including metropolitan (Newcastle) and regional areas (Tamworth). However, the sample recruited was mostly homogeneous in term of breakfast habits, as they were mostly early Habitual Breakfast Eaters, and in terms of occupation, were mostly professional, paraprofessional, managers or administrator, administrative assistants, sales or personal service workers. It is possible that because the recruitment fliers indicated that the study was investigating breakfast and its relationships with health parameters, this biased the recruitment to those subjects who habitually consumed breakfast and were interested in the health benefits of this behaviour. Moreover, the relatively small sample does not allow the results to be generalized with confidence to the wider Australian male population. Furthermore, the evidence reported in this study is based on self-reported data, however many questions, including the demographic and breakfast frequency questions, have been widely used in Australia by large national surveys such as ALSWH [14], Australian Bureau of Statistics (ABS) in 2011 [15] or NNS in 1995 [13], and therefore, these questions can be considered well established methods to collect this self-reported information.

## Conclusions

This study found that in this sample of Australian men the majority consume breakfast most days of the week, and do so before 8 am. A majority of Australian men less than 45 years old were found to be Habitual Breakfast Consumers. Cereal, milk and fruit were the most common breakfast foods consumed for breakfast in this study. While this was also the case in the 1995 National Nutrition Survey (NNS), there appear to be some differences in the proportion contributions for this study. Specifically, there is an indication that the proportion of men eating fruit and breakfast cereal consumption may have increased. However, we must recognise the inherent differences in data collection and design between the studies and not draw any strong conclusions about changes in breakfast consumption over time.

## Supplementary information

**Additional file 1.** Survey Questions. This file describes the questions of the survey

**Additional file 2.** Supplementary Data File. This file contains the raw data presented in this paper

## Data Availability

All data generated or analysed during this study are included in this published article (Tables 1,2,3) and its supplementary information files (Additional file 1 & Additional file 2).

## Abbreviations

ABS: Australia Bureau of Statistics
ALSWH: Australian Longitudinal Study of Women’s Health
ATSI: Aboriginal or Torres Strait Islander
BMI: Body Mass Index
EBE: Early Breakfast Eaters
FFQ: food frequency questionnaire
HBE: Habitual Breakfast Eaters
HBS: Habitual Breakfast Skippers
LBE: Late Breakfast Eaters
NA: Not Applicable
NNS: Australian National Nutrition Survey
NSW: New South Wales
OBE: Occasional Breakfast Eaters
WHO: World Health Organisation
