# Supplementary material for: Breakfast consumption habits of Australian men participating in the “Typical Aussie Bloke” study
Source: BMC Nutr. 2020 Jan 7;6:1. doi: 10.1186/s40795-019-0317-4 (PMC7050778; doi:10.1186/s40795-019-0317-4)
Supplement: Supplementary file 1 — Additional file 1. Survey Questions. This file describes the questions of the survey [file 40795_2019_317_MOESM1_ESM.docx]

**Appendix 1**

**Survey Questions**

1. **Please indicate the time you usually wake up on weekdays.**

| Midnight to 5.00 am |
| --- |
| 5.01 to 6.00 am |
| 6.01 to 7.00 am |
| 7.01 to 8.00 am |
| 8.01 to 9.00 am |
| 9.01 to 10.00 am |
| 10.01 to 11.00 am |
| 11.01 am to noon |
|  |

*If participants do not answer to this question, this message will appear:* This question requires an answer.

1. **Please indicate the time you usually wake up on weekend days.**

| Midnight to 5.00 am |
| --- |
| 5.01 to 6.00 am |
| 6.01 to 7.00 am |
| 7.01 to 8.00 am |
| 8.01 to 9.00 am |
| 9.01 to 10.00 am |
| 10.01 to11.00 am |
| 11.01 am to noon |

1. **Please indicate the time you usually have the first meal of the day on weekdays.**

| Midnight to 5.00 am |
| --- |
| 5.01 to 6.00 am |
| 6.01 to 7.00 am |
| 7.01 to 8.00 am |
| 8.01 to 9.00 am |
| 9.01 to 10.00 am |
| 10.01 to11.00 am |
| - 1. m to noon |
|  |

1. **Please indicate the time you usually have the first meal of the day on weekend days.**

| Midnight to 5.00 am |
| --- |
| 5.01 to 6.00 am |
| 6.01 to 7.00 am |
| 7.01 to 8.00 am |
| 8.01 to 9.00 am |
| 9.01 to 10.00 am |
| 10.01 to11.00 am |
| 11.01 am to noon |
|  |

1. **How many days per week do you usually have something to eat for breakfast?**

Rarely or never

1 to 2 days

3 to 4 days

5 or more days

Don't know/varies

1. **How many times per week do you usually have these foods for breakfast?**

|  | Times per week |
| --- | --- |
| Fruits |  |
| Toast |  |
| Butter and/or margarine |  |
| Spread (e.g. jam, honey, peanut butter, Nutella, vegemite, etc.) |  |
| Cereals like All bran |  |
| Cereals like Sultana Bran^TM^, FibrePlus^TM^, BranFlakes^TM^ |  |
| Cereals like Weet bix^TM^ ,Vita Brits^TM^, Weeties^TM^ |  |
| Cereals like CornFlakes, Nutrigrain^TM^ , Special K^TM^ |  |
| Cereals like Porridge |  |
| Cereals like Muesli |  |
| Milk for cereal |  |
| Yogurt |  |
| Eggs |  |
| Bacon |  |
| Beans |  |
| Pancake/crepes |  |
| Other foods (please specify): |  |

1. **How many times per week do you usually have these beverages for breakfast?**

|  | Times per week |
| --- | --- |
| Coffee |  |
| Tea |  |
| Milk on its own |  |
| Hot chocolate milk/ milo |  |
| Juice |  |
| Smoothies |  |
| Other beverages (please specify): |  |

1. **What is the main reason you eat breakfast? (Choose only one option)**

It gives me energy

I want to lose weight

It helps prevent me from getting hungry before lunchtime

I enjoy it

It helps me to wake up

It is what I always do

I am hungry

Eating breakfast makes it easier to control my weight

Others reasons

If other reasons was selected, please specify:

1. **On days that you do not have breakfast, what is the reason?**

Not enough time

I do not feel like eating first thing

I want to lose weight

Hung over

I have a cigarette instead

I do not have any food in the house

I do not have enough money to have breakfast

I rarely/never don’t eat breakfast

Other reasons

If other reasons was selected, please specify:
